# Between Securitisation and Neglect: Managing Ebola at the Borders of Global Health

**DOI:** 10.1017/mdh.2017.6

**Published:** 2017-04

**Authors:** Mark Honigsbaum

**Affiliations:** School of History, Queen Mary University of London, Mile End Road, E1 4NS, UK

**Keywords:** Ebola, WHO, Emerging infectious diseases, Biosecurity, Fear, Rumour

## Abstract

In 2014 the World Health Organization (WHO) was widely criticised for failing to anticipate that an outbreak of Ebola in a remote forested region of south-eastern Guinea would trigger a public health emergency of international concern (pheic). In explaining the WHO’s failure, critics have pointed to structural restraints on the United Nations organisation and a leadership ‘vacuum’ in Geneva, among other factors. This paper takes a different approach. Drawing on internal WHO documents and interviews with key actors in the epidemic response, I argue that the WHO’s failure is better understood as a consequence of Ebola’s shifting medical identity and of triage systems for managing emerging infectious disease (EID) risks. Focusing on the discursive and non-discursive practices that produced Ebola as a ‘problem’ for global health security, I argue that by 2014 Ebola was no longer regarded as a paradigmatic EID and potential biothreat so much as a neglected tropical disease. The result was to relegate Ebola to the fringes of biosecurity concerns just at the moment when the virus was crossing international borders in West Africa and triggering large urban outbreaks for the first time. Ebola’s fluctuating medical identity also helps explain the prominence of fear and rumours during the epidemic and social resistance to Ebola control measures. Contrasting the WHO’s delay over declaring a pheic in 2014, with its rapid declaration of pheics in relation to H1N1 swine flu in 2009 and polio in 2014, I conclude that such ‘missed alarms’ may be an inescapable consequence of pandemic preparedness systems that seek to rationalise responses to the emergence of new diseases.

On 24 March 2014, the deputy director of the World Health Organization’s Emergency Response team in Geneva held an urgent teleconference with the regional director of the WHO’s Africa office (AFRO) to discuss the ongoing outbreak of Ebola in a remote forested region of south-eastern Guinea – an outbreak which, at that point, had resulted in 111 clinically suspected cases and 79 deaths. Preliminary sequencing suggested the deaths were due to *Zaire ebolavirus* – a species of Ebola associated with very high mortality in previous outbreaks in the Democratic Republic of the Congo (DRC) and Gabon. Even more alarming, the outbreak appeared to be spreading rapidly and had already caused the deaths of four health care workers, suggesting that infection controls that should have been in place in hospitals and clinics in Guinea were not working. Concerned that the virus might soon cross the border into Sierra Leone and Liberia, resulting in an unprecedented outbreak in three countries in West Africa simultaneously, WHO officials recommended upgrading the outbreak to ‘an internal grade 2 emergency’.^1^

The grading system, established a few years earlier as part of the WHO’s Emergency Response Framework (ERF), was a way of triaging scarce resources and rationalising the WHO’s response to outbreaks of emerging infectious diseases (EIDs) that were thought to constitute ongoing threats to global health security.^2^ Under the provisions of the 2005 International Health Regulations (IHR), outbreaks caused by novel strains of influenza, such as the H5N1 bird flu virus, and epidemics of poliomyelitis and Severe Acute Respiratory Syndrome (SARS) automatically mandated the declaration of a public health emergency of international concern (pheic). By contrast other EIDs, such as Ebola, were subject to more nuanced judgements by officials in Geneva in which the crucial test was whether an outbreak might have ‘serious health impacts’ and pose a ‘significant risk of international spread’.^3^ Even then, the declaration of a pheic was not a foregone conclusion. Instead, officials in Geneva were required to follow a step-by-step flow chart and directive algorithms in order to determine at what point an outbreak of Ebola or some other EID called for a higher grade response from the WHO and became sufficiently serious to override the rights of member states to the self-determination of health risks within their borders.

Upgrading the outbreak to grade 2 would enable the WHO to strengthen its emergency support at country level and dispatch experts to Guinea-forestière to advise on outbreak control, but, crucially, it would not risk alarming international business or Guinea’s trading partners since the re-grading would signal that the WHO considered the outbreak had ‘moderate public health consequences’. Three months later this was still the WHO’s position. Treating Ebola as an international emergency ‘could be seen as a hostile act…and may hamper collaboration between the WHO and affected countries’, warned Keiji Fukuda, the WHO’s assistant director general for Health, Security and Environment, in an internal briefing note to the WHO director general, Margaret Chan, in June 2014. ‘This outbreak must be considered as a sub-regional public health issue’.^4^ Sylvie Briand, director of the WHO’s Pandemic and Epidemic Diseases Department, concurred. ‘I don’t think declaring a pheic will help fight the epidemic at this stage’, she emailed a colleague on 4 June. ‘The problem with declaring a pheic is that one has to make recommendations and these risk hurting the country without helping public health…I see using the IHR as a last resort’.^5^ As a result, it not until late July that Chan upgraded the emergency to grade 3 and it was not until 8 August that, bowing to international pressure and concerns that, in the words of Médecins Sans Frontières (MSF), the outbreak in West Africa was ‘totally out of…control’, Chan finally declared Ebola a pheic.^6^

In the weeks and months that followed Chan’s decision, the WHO’s prevarication during the early stages of what was, by then, the largest Ebola outbreak in history would come to be regarded as fatal, and the internal emails and documents, would become primary exhibits in inquests into the WHO’s failure to launch a more robust and rapid response to the epidemic – inquests that would lay the blame on a ‘vacuum of leadership’ in Geneva and the inability or unwillingness of political appointees in AFRO to confront politicians in the affected countries.^7^ By contrast, global health and international security experts were rather kinder to the WHO, arguing that its failure to take more decisive action in the initial weeks and months of the outbreak should be seen in the context of structural restraints on the international organisation and its precarious financial and political position. In particular, they pointed to swingeing budget cuts, which curtailed the WHO’s capacity to mount a surge response, and the traditional autonomy enjoyed by member states for setting health priorities and taking strategic decisions about outbreaks within their borders. It is argued that it was only later, when Ebola overwhelmed core capacities in the tri-border region of West Africa threatening the security of other countries, that the WHO abandoned this ‘delegated’ model of authority and became more proactive.^8^

This article takes a different approach. Rather than seeking to blame or exonerate the WHO, my aim is to understand how and in what ways medical and other forms of expertise were brought to bear – or not brought to bear – on the ‘problem’ of Ebola. By problem, I mean not merely the messy materiality of the virus, but the discursive and non-discursive practices that also produced Ebola as an EID and a problem for global health security.^9^ These biosecurity problematisations are by no means unique to Ebola. From HIV/AIDS, to SARS, bird flu, and, most recently, the Zika virus, there is a rich literature on the way that modern biosurveillance regimes and systems of global health governance produce EID risks in historically and ontologically distinct ways.^10^ However, with Ebola and the WHO’s response to the 2013–16 epidemic in West Africa we are presented with a stark example of the limitations of these assessment systems and the flaws in biosecurity regimes and pandemic triage systems built on such entanglements of knowledge and practice.

Drawing on oral interviews with key international health responders and scientific actors in the epidemic, the documents obtained by Associated Press, and the historical literature on Ebola, I argue that the WHO’s failure had at least as much to with shifting medical and security constructions of the virus as it did with the organisation’s leadership and structural factors.^11^ Tracing these security constructions back to the accidental outbreak of Ebola that occurred in 1989 at a primate quarantine facility near Washington DC, I show how Ebola has undergone multiple transformations in its relatively short career as an EID and a site of public health concerns – one that has seen it fluctuate from a potential biowarfare agent to a neglected tropical disease, and back again. These constructions are further complicated by the fracturing of Ebola’s identity at a molecular level as successive outbreaks have led to the steady addition of new Ebola viral subtypes, each with its own distinctive nomenclature and position on Ebola’s phylogenetic tree, prompting disputes over the correct name of the genus and its subspecies and speculation about variations in virulence and the virus’s mutability.^12^ Indeed, one of the tragedies of the West African epidemic is that by the time it was recognised that the outbreak in Guinea-forestière was due to *Zaire ebolavirus* (EBOV) – the same strain of Ebola that had been associated with earlier outbreaks with high mortality in central Africa – the virus had spread to Sierra Leone and Liberia and was already widely dispersed in the tri-border region.^13^

My analysis also highlights the limitations of pandemic preparedness systems that seek to triage or grade risk responses to EIDs that are thought to constitute differential threats to global health security, without regard for the way that these epidemic and pandemic threats are themselves the products of discourses about EIDs. This proved particularly fatal in the case of Ebola, I argue, because of the way these discourses fluctuated between securitisation and neglect. As Paul Farmer pointed out in 1996 in his call for a critical epistemology of EIDs, the EID concept has been highly effective in unlocking research funding and galvanising large government bureaucracies, particularly in Washington DC where EIDs have long been framed as a threat to the security of the United States. At the same time, he writes, the concept carries ‘complex symbolic burdens’ that obscure other ways of thinking about and responding to these outbreaks.^14^ Nunes makes a similar point when he argues that by designating the West African outbreak a pheic using the EID framing, the WHO automatically inscribed Ebola as a security problem and an object of short-term crisis management, thereby obscuring the social, economic and environmental dimensions of the outbreak and the ways in which the virus had ‘been allowed to remain a problem’ for some regions of Africa for more than forty years.^15^ Finally, I argue that the epidemic coincided with the relegation of Ebola as an international health priority, a shift that was reflected in management systems for triaging between different EID risks and assessing when an Ebola outbreak required the declaration of a pheic. The result was that unlike other pathogens named in the IHR (2005), in early 2014 Ebola was not considered an urgent threat to global health security. Nor, despite considerable investment in experimental drugs and vaccines in the decade leading up to the outbreak, was Ebola considered a high public health priority. Ebola’s shifting medical and cultural identity also helps explain why, once it became clear that the virus was spreading faster and more widely than anticipated, the WHO and other agencies became paralysed by fear, furthering delaying the deployment of responders to the Ebola zone.

In § 1, I review the history of Ebola and its emergence as a site of biosecurity concerns. Juxtaposing these securitisation discourses with the evolution of scientific knowledge of the virus, its taxonomy and epidemiology, I argue that by 2014 Ebola was no longer regarded as a highly pathogenic ‘hot virus’ and potential biothreat so much as a viral haemorrhagic fever (VHF) endemic to central Africa, and which, it was thought, could be contained by basic hygiene measures and the rapid deployment of technical experts to the foci of outbreaks. In § 2, I turn to the regulatory mechanisms and managerial procedures for the governance of Ebola and other generic biothreats. Focusing on the operation of the IHR (2005) and the ERF, I argue that the West African outbreak revealed flaws in risk triaging systems put in place after the ‘false alarm’ of the H1N1 swine flu outbreak in 2009. The result was that Ebola was relegated to the fringes of global health security just at the moment when the virus was crossing international borders and triggering urban outbreaks for the first time. In § 3, drawing on the work of medical anthropologists, I examine the role of rumour, panic and fear in mediating responses to the outbreak, showing how the framing of Ebola as a hot virus and security threat confused communities in the affected countries, fuelling distrust of international health responders and the biomedical messaging. At first, the terrifying popular image of Ebola also hampered the emergency response. It was only when Ebola threatened an epidemic beyond West Africa and the extent of the death and suffering within the tri-border zone became too visible to ignore, that, I argue, both the affected communities and leading global health actors responded with alacrity. Contrasting the delay over Ebola with the WHO’s rapid response to the emergence of SARS and its similarly rapid declarations of public health emergencies for swine flu in 2009 and polio in 2014, I conclude that the WHO’s ‘failure’ flowed both from Ebola’s shifting medical identity and from the organisation’s attempt to rationalise its response to novel emergence events – events that are unpredictable and unknowable.

## 1 A Perfect Parasite

There is little doubt that Chan and other senior WHO officials were too slow to realise the extent to which the outbreak in Guinea-forestière posed a wider epidemic threat or that the virus would rapidly spread across borders, overwhelming the health capacities of Guinea, Liberia and Sierra Leone – three of the poorest countries in West Africa – to infest three cities simultaneously (Conakry, Monrovia, Freetown). However, if so, then they were not alone. In 2014 most experts considered Ebola a disease of remote forested regions of equatorial Africa. Indeed, the key observation from the previous eleven large outbreaks of Ebola was that such outbreaks tended to be self-limiting and that it was only when the virus entered a nosocomial setting with low standards of hygiene and sanitation that it became a source of epidemic amplification. This was particularly the case where hospital workers failed to employ appropriate barrier-nursing techniques and ignored standard hygiene measures, such as bans on the reuse of syringes and hypodermic needles.^16^ As David Heymann, the head of Chatham House’s Centre on Global Health Security and a veteran of several Ebola outbreaks in Africa, explained at a meeting at Chatham House in May 2014 called to ‘measure the risk’ of the outbreak in Guinea, with proper hygiene controls and barrier nursing Ebola was ‘it’s own worst enemy…[because] it’s too virulent to sustain its transmission’.^17^ Pierre Rollin, a French-born viral haemorrhagic fever (VHF) expert at the Centers for Disease Control and Prevention (CDC) in Atlanta, was similarly unconcerned by the outbreak in Guinea. Dispatched to Conakry in March to build bridges with the Guinean government and assess the situation on the ground, Rollin returned to Atlanta in May believing that cases had peaked and that the virus would rapidly burn itself out. ‘It looked like, smells like, tastes like regular outbreaks in previous areas,’ he recalled thinking to himself at the time.^18^ This was despite his awareness of a 1982 paper in the *Annales de L’Institut Pasteur* which had found that people treated for Lassa fever at a hospital in Lofa county, Liberia, just across the Guinean border, carried antibodies to both Lassa and Ebola, suggesting the filovirus had been present in the region for some time and that earlier Ebola infections might have been missed.^19^

The framing of Ebola as a ‘hot virus’ owes much to the work of *New Yorker* journalist Richard Preston and the simultaneous outbreaks that occurred in 1976 at a Catholic mission in Yambuku in the DRC (Democratic Republic of Congo, former Zaire) and Sudan. Although Ebola outbreaks had most likely occurred before in other parts of central Africa, this was the first to come to the attention of international health responders and result in the identification of the filovirus.^20^ In his 1994 bestselling book *The Hot Zone*, Preston had focused on the most lurid and visually shocking symptoms of Ebola, such as the way that in the last stage of illness patients sometimes ‘bled out’, leaking blood and haemorrhagic fluids from their eyes, noses and intestines. Even though such symptoms occur in only about half of Ebola cases they helped fix the idea that Ebola was, as Preston put it, ‘a perfect parasite…[that] transforms virtually every part of the body into a digested slime of virus particles’.^21^ Through the imaginative use of flypapers marked with biosafety hazard warnings, Preston also reinforced the impression of Ebola as a potential biowarfare agent, one that could emerge from the jungles of Africa, or the laboratory of a deranged terrorist, to threaten global health security at any time. As King points out, this framing of Ebola owes much to an outbreak that occurred in 1989 at a primate quarantine facility in Reston, Virginia, just across the Potomac from Washington DC.^22^ Triggered by an Ebola subtype harboured by monkeys imported from the Philippines, the *Reston ebolavirus* spread rapidly between primates kept in different rooms in the quarantine facility, raising the prospect that Ebola was capable of aerosol transmission and prompting scientists to euthanise the monkeys and decontaminate the premises. Although the outbreak resulted in four subclinical infections in laboratory workers, in the event no humans died and it was concluded that concerns that Ebola might be a viable aerosol were overblown. Nevertheless, the proximity of the outbreak to the US capital underlined Ebola’s threat to US national security, resulting in its selection for a war-game exercise at a meeting of the American Society of Tropical Medicine and Hygiene in Honolulu the same year.^23^

More significantly, the Reston incident contributed to Ebola appearing alongside an iconic list of EIDs highlighted in an influential report on ‘Emerging Infections’ by the Institute of Medicine (IoM). Prior to 1992, epidemiologists had referred to Ebola as a ‘new’ infectious disease.^24^ Its inclusion in a list that contained HIV, the cause of the recent AIDS pandemic, immediately elevated its political status. According to the IoM, EIDs were a special group of new diseases associated with ‘clinically distinct conditions whose incidence in humans has increased’ and for which ‘emergence may be due to the introduction of a new agent, to the recognition of an existing disease that has gone undetected, or to a change in the environment that provides an epidemiologic “bridge” ’.^25^ Interestingly, at this point it was not clear how many of these criteria applied to Ebola as its presumed animal reservoir was, and still is, unknown, making the role of environmental change in the transfer of the virus from animals to human hosts speculative. Indeed, the virus’s inclusion in this list appears to have been largely a result of the Reston incident and the way that the outbreak revealed the risk that the commercial trade in research primates posed for the carriage and spread of potentially deadly rain forest pathogens across international borders – a theme that would assume increasing importance in books on emerging infections published subsequent to the IoM report.^26^ It is also worth noting that the authors of the report took pains to challenge the assumption that such emergence events were always or largely due to spontaneous mutations in animal viruses and their adaptation to new hosts. On the contrary, the authors pointed to the significance of several other factors, including changing human demographics and behaviour, economic development and land use, and the breakdown of public health measures.^27^ All three of these would prove highly relevant to the emergence of Ebola in West Africa in 2013–16.^28^

Unfortunately, these perspectives would be obscured when, after a nearly twenty-year hiatus, Ebola emerged for a second time in 1995, causing an outbreak in Kikwit, a city in the DRC. Coming in the wake of the popular success of *The Hot Zone*, the outbreak sparked tremendous press interest, transforming the emergency response into a media circus.^29^ The result was that it was Preston’s and not the IoM’s more measured assessment of the risks posed by EIDs that shaped the view of Ebola in policy circles in Washington in the mid to late 1990s, resulting in what one virologist described as a ‘shower of funding’ for vaccine research.^30^ The highpoint of this trend came in around 2008/09 when, in the wake of the 2002 anthrax attacks and the Bush administration’s war on terror, the National Institute for Allergy and Infectious Diseases (NIAID) was awarded $4.7 billion for research into vaccines for Ebola and other VHFs. However, by 2013, following the conclusion of the war in Iraq, NIAID’s funding had fallen to $4.3 billion, the same level as in 2009.^31^ In the absence of compensating investment from the private sector, this funding freeze was later blamed for the fact that when Ebola re-emerged in West Africa in 2014 there were no licensed vaccines or drugs ready for deployment to the Ebola zone.^32^

King and others have argued that the IoM report marked the moment when infectious diseases began to be regarded as security threats, rather than merely as threats to health and free trade, and their surveillance became the object of a new paradigm of global health governance.^33^ Tracing this paradigm shift is beyond the scope of this paper but a key moment came in 1994 when the WHO established the Division of Emerging and Other Communicable Diseases Surveillance to improve surveillance of EIDs and co-ordinate relations with NGOs, expert advisors and collaborating centres on the ground. This was followed, six years later, by the establishment of the WHO’s Global Outbreak and Response Network (GOARN) to enable the rapid mobilisation of knowledge and expertise relating to EIDs.^34^ However running alongside these securitisation discourses, was a very different construction of Ebola, one that configured it as just another haemorrhagic fever endemic to the central African rainforest. By 2004 genomic analysis had demonstrated that while the genes of Ebola subtypes were sufficiently different from one another to warrant their own place on its phylogenetic tree, overall the virus showed a high degree of genomic stability.^35^ According to some experts, this suggested that concerns that Ebola might mutate into an ‘Andromeda strain’ were overblown.^36^ Rather, the genetic differences suggested that each subtype had emerged from an animal reservoir in an area geographically isolated from other subtypes in or near the site where transmission to humans and non-human primates had first occurred.^37^ Moreover, while the Yambuku outbreak had seen case mortality rates as high as 90 per cent, in Kikwit the mortality rate had been 78 per cent, while in an outbreak in Gabon the following year the mortality rate had been 57 per cent.^38^ Clearly, for all the concerns about Ebola’s virulence, infection was not an automatic death sentence. Even in large outbreaks, such as had occurred in Gulu, Uganda, in 2000–01, or in Gabon and Congo in 2001–02, casualties had numbered in the hundreds. Indeed, in the two dozen outbreaks of Ebola prior to 2014, the virus had been responsible for a total of just 2200 infections and 1500 deaths.^39^ Compared to AIDS, or more widely prevalent tropical diseases such as malaria, this made Ebola more of a nuisance than an urgent public health threat, one that, it was thought, could be managed through close epidemiological and laboratory surveillance, coupled with the rapid deployment of experts trained in the containment of VHFs to the foci of outbreaks. As Daniel Bausch, a haemorrhagic fever specialist based at Tulane University, and Pierre Rollin put it in a 2004 review of the lessons learned from the Ebola outbreaks in Uganda, Gabon and Congo: ‘In most instances, what likely converts low-level endemic transmission to an epidemic is nosocomial amplification in the context of the incomplete health care infrastructure commonly found in tropical Africa in recent decades’. The problem, they thought, was that all too often ill-equipped ‘Western-style hospitals’ staffed by doctors and nurses with little experience of Ebola had created a ‘fertile ground for epidemic EHF [Ebola Haemorrhagic Fever]’. But rather than adopt a more critical epistemology of EIDs – one that took seriously the other factors in emergence identified in the IoM report, such as broken health systems and changing land use patterns – they simply called for the WHO to adapt GOARN to the technical exigencies of Ebola. Even as Bausch and Rollin bought into this emergency response model, however, they could not help but express doubts about the WHO’s ability to rapidly detect Ebola outbreaks and judge whether they necessitated the activation of GOARN. Crucially, this had as much to do with the shifting construction of Ebola as it did with the WHO’s management expertise and choices about the allocation of resources. ‘EHF poses a unique problem – too rare to merit a large investment in each African community, yet too virulent to completely ignore,’ they concluded. The result was a ‘predicament’ in which the international community was faced with ‘attempting to balance the inevitable preponderance of false alarms across the African continent with readiness for rapid action when the real emergency occurs’.^40^

## 2 Triaging Risks to Biosecurity

Though the rarity of Ebola outbreaks coupled with the virulence of the virus presented a problem for the governance of Ebola, it should not have been insuperable. As the above review demonstrates, the measures needed to control and limit the spread of Ebola were well known to VHF experts. Moreover, through GOARN and agencies such as the CDC, there was plenty of expertise that could be brought to bear on the problem of an Ebola epidemic. Indeed, during the SARS outbreak the WHO had won plaudits for its rapid mobilisation of international experts, thereby averting what could have been a much worse crisis.^41^ In the case of Ebola, however, GOARN does not appear to have functioned in the way it was intended. Following the confirmation on 22 March 2014 of EBOV in blood samples forwarded by MSF from Guéckédou to the Institut Pasteur in Lyons, the WHO dispatched an interdisciplinary team equipped with diagnostics and protective equipment to Guinea-forestière. By mid-April Geneva was co-ordinating some sixty-five foreign epidemic experts in Guinea, including a team from the CDC.^42^ Those numbers doubled as Ebola spread to Sierra Leone and Liberia, but by mid-May, as people presenting with Ebola at clinics and treatment centres declined, GOARN was wound down. Kamradt-Scott points out that in previous Ebola outbreaks the WHO had dispatched an average of just two to five experts to the foci of Ebola epidemics and that it is unfair to blame the WHO for not mobilising a more comprehensive technical response in 2014 given that the organisation’s ‘primary task has always been to coordinate, only assisting governments upon request’.^43^ Moreover, when in June epidemiological evidence pointed to a resurgence of infections in the tri-border region, the WHO rapidly increased the deployment of personnel to the Ebola zone. The result was that by the conclusion of the epidemic the WHO had deployed 1000 experts to West Africa.^44^ However, David Heymann, the WHO’s former Executive Director for Communicable Diseases and one of the architects of GOARN, believes that officials in Geneva could and should have done more. ‘For some reason, after the initial response was made, GOARN and the partners, including the Center for Disease Control and Prevention in the US, thought there was no longer a concern and so they left,’ Heymann said. ‘Normally, what GOARN does is waits until they’ve been able to verify for two full incubation periods, a time period of in this case 42 days’.^45^

Why this did not happen in Guinea is unclear. One explanation suggested by the emails obtained by AP is that a key factor may have been the reluctance of the Guinean government to acknowledge the full extent of the outbreak, coupled with the understandable concern of WHO officials in Geneva that declaring a pheic might trigger negative measures, such as border closings and travel bans, that could hinder business and the dispatch of health workers to the Ebola zone.^46^ However, another is that in the previous two decades Ebola had undergone what Lakoff calls a ‘conceptual mutation’ so that by 2014 the virus was no longer seen as ‘the source of a potentially catastrophic global epidemic’. Instead, Lakoff suggests that experts approached the West African outbreak with ‘relative confidence’, believing that Ebola could be ‘managed via localized humanitarian care combined with straightforward public health techniques’. In short, Ebola was no longer ‘the novel and fearsome virus that helped spark attention and resources to the phenomenon of “emerging infectious disease” in the late 1980s and early 1990s’.^47^

In this section I would like to take up this insight and relate it to the regulatory mechanisms and managerial procedures for the governance of infectious diseases listed in the IHR (2005) and covered by the ERF. Ever since the 2003 SARs outbreak, the central challenge facing global health authorities has been to detect and contain outbreaks of novel pathogens before they spark international emergencies, without compromising the sovereignty of states over the regulation of epidemic risks. Under the IHR (2005), which revised and replaced the earlier 1969 regulations, this was to be achieved by encouraging WHO member states to take greater responsibly for the ongoing management of disease threats within their borders, by increasing surveillance and risk assessment at ports of entry and exit, and by strengthening core health capacities. At the same time, the regulations provided a framework for co-ordinated and proportionate responses to urgent disease threats that were beyond countries’ core capacities and which the WHO deemed presented a significant risk of regional or international spread. In such circumstances, affected countries would gain access to GOARN, enabling the WHO to dispatch up to 300 technical and operational experts to the site of an outbreak and feed information back to Geneva. Once apprised of this information, the WHO’s director general would then be in a position to determine whether a country’s response was adequate or whether there was risk of international spread, in which case the director general had the authority to declare a pheic.^48^

To the extent that the revised regulations encouraged states to become active managers of the health of their populations they can be seen as part of what Elbe calls a late twentieth-century trend towards the ‘medicalisation of security’.^49^ Taking up these insights, Bingham and Hinchcliffe suggest that one of the characteristics of modern biosecurity regimes is that they seek to regulate the flow of ‘good’ things (trade, tourists, political refugees) across territorial boundaries, while simultaneously limiting the flow of potential security threats (pathogens, terrorists, economic migrants). Underpinning these biosecurity regimes are both active and passive forms of surveillance, with the latter, in theory, acting as a check or failsafe on the former.^50^ Such regimes are far from perfect, however. Furthermore, to the extent that they require state and non-state actors to make nuanced judgements as to when an outbreak constitutes a risk of ‘international spread’ they are open to interpretation. This was precisely the dilemma that faced Chan in 2009 when the sudden emergence of a new swine influenza A virus in Mexico raised the prospect of a global flu pandemic. In response, Chan convened an advisory committee of experts, known as the Emergency Committee, to weigh the evidence and declare a pheic. However, that decision backfired when it became clear that the H1N1 virus was no more severe than a seasonal flu and that many committee members had associations with vaccine manufacturers – the principal beneficiaries of Chan’s announcement.^51^

**Figure 1:**
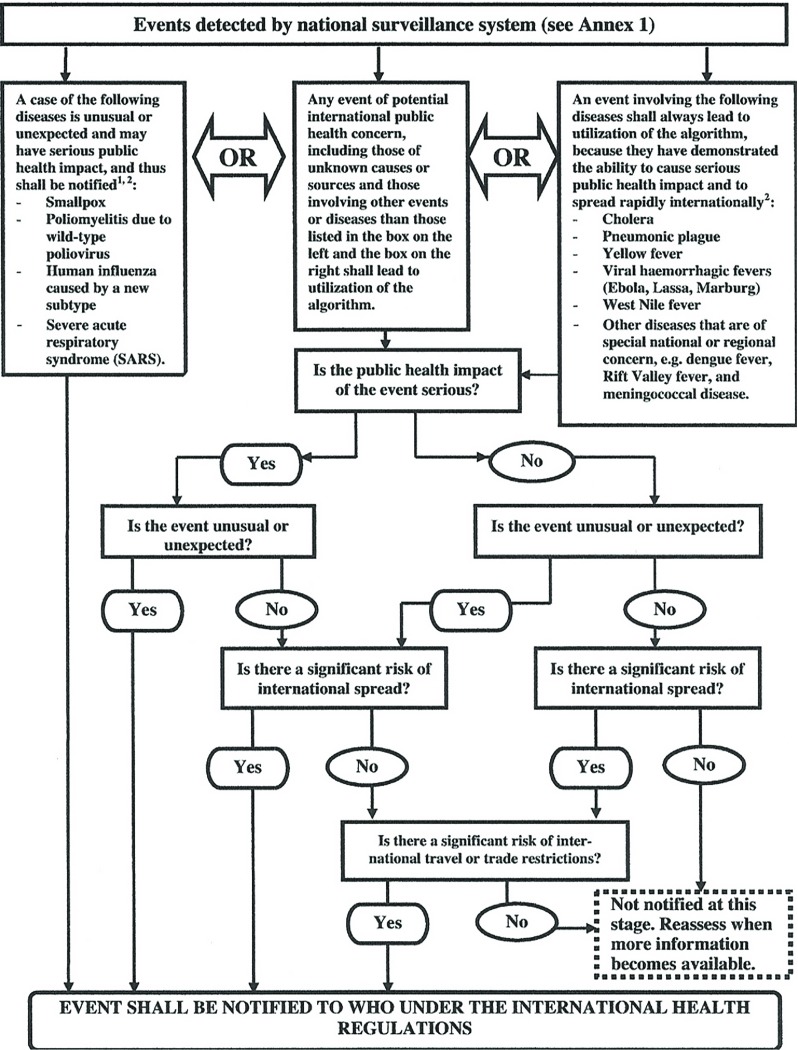
‘Decision Instrument for the assessment and notification of events that may constitute a public health emergency of international concern’. Source: Reprinted from: WHO, International Health Regulations (2005), Annex 2, 43, http://www.who.int/ihr/publications/9789241596664/en/ (accessed 9 January 2017). Credit: WHO

The ERF was an attempt to introduce transparency into these managerial processes and clarify the WHO’s leadership obligations at a time when global emergencies and the consequent demands on the WHO’s time were increasing.^52^ To allocate the WHO’s limited resources in the most rational way, while meeting its regulatory responsibilities under the IHR and as a member of the Inter-Agency Standing Committee (IASC) for humanitarian emergencies, the ERF delegated decision-making to a Global Emergency Management Team (GEMT). It was the GEMT’s job to conduct risk assessments and decide when an event with public health consequences required a response from the WHO. Once the ERF had been triggered, it was for committee members and officials in Geneva, together with the appropriate country office, to decide to what extent the WHO was required to provide emergency support, the urgency of the response and the appropriate grading level. However, this grading process was separate from other international emergency classification procedures, including the procedures laid out in the IHR (2005), and was the responsibility of a separate managerial team within the WHO headed by Bruce Aylward, assistant director-general for polio and emergencies. Infectious disease outbreaks other than polio were the remit of Keiji Fukuda and were subject to another set of procedures. These procedures were laid out in an annex to the IHR (2005) and included a ‘decision instrument’ with a flow chart for deciding when an event might require the declaration of a pheic (see Figure 1).

Crucially, the only diseases that, according to this chart, required automatic notification to the WHO were smallpox, poliomyelitis due to wild-type poliovirus, human influenza due to the emergence of a new subtype, and SARS. By contrast, outbreaks of Ebola and diseases such as cholera, plague and yellow fever, were subject to an algorithm in which officials were asked to judge, firstly, whether the outbreak constituted a ‘serious’ threat to public health, and second, whether the event was ‘unusual and unexpected’. Only if these first two conditions were met, could officials go on to ask whether there was also ‘a significant risk of international spread’ and ‘a significant risk of international travel or trade restrictions’, in which case they were required to notify the WHO.^53^ By definition, then, there had to be a risk of an outbreak transcending a national border before the alert level could be elevated and WHO officials would consider a pheic.

Of course, it would be naïve to suggest that such risk triage systems are only guided by administrative processes; political and bureaucratic priorities surely also play a role, as does investment in pre-existing health programmes. To see how such considerations can influence the WHO’s assessments of infectious disease risks one only has to contrast Chan’s decision not to raise the alert level for Ebola with her decision in May 2014 to declare a pheic in relation to polio. Unlike Ebola, polio is a highly eradicable disease. An easy to administer oral vaccine has existed since the 1950s and although mutations in vaccinated individuals can lead to the emergence of new vaccine-derived strains of the disease these can be neutralised by immunisation with killed polio vaccines. Indeed, since the launch of the Global Polio Eradication Initiative in 1988 the world has seen dramatic declines in the incidence of polio. By 2013 this effort had led to the near cessation of polio transmission. However, in 2014 there was a resurgence of polio in Afghanistan and Pakistan. In addition, outbreaks were recorded in six previously polio-free non-endemic countries, including Somalia, Equatorial Guinea, Syria and Iraq, due to the spread of the virus across international borders. Interestingly, these were states that, like Sierra Leone and Liberia, had been plagued by war and civil conflicts – some of which were ongoing – and which had left the countries with severely compromised immunisation services and weakened health systems. However, the key reason the Emergency Committee gave for the declaration of a pheic was not that without outside intervention these states would be unable to prevent the re-infection of their populations, but because ‘if unchecked, this situation could result in failure to eradicate globally one of the world’s most serious vaccine-preventable diseases’.^54^ No such programmatic goals or bureaucratic interests were threatened by the sudden emergence of EBOV in Guinea-forestière, however. Nor, when Ebola began to spread across the border to Sierra Leone and Liberia and calls mounted for the WHO to take more robust action, did the agency alter its policy of maintaining a watching brief. Had there been a vaccine or an arsenal of drugs ready to be deployed, as had been the case during the 2009 swine flu pandemic, things might have been different, but by 2013 Ebola research had ground to a halt.^55^ Nor, despite repeated calls for a network of reporting laboratories in Africa equipped with emergency diagnostic tests for Ebola and other EHFs, had such calls been heeded.^56^ In short, Ebola had become a neglected disease.

## 3 The Dog that Didn’t Bark

If the paradigm that prevailed in medical and public health circles immediately prior to the outbreak was that Ebola was a self-limiting disease that could be contained with relatively low-tech interventions, by April 2014 people were being reminded of Ebola’s other, more alarming identity as a ‘hot virus’. Initially, this Ebola was only apparent to the early responders on the ground. However, as the virus spread across the border to Sierra Leone and Liberia and patients began presenting with symptoms of haemorrhagic fever at hospitals in Freetown and Monrovia, this Ebola became increasingly visible, paralysing the authorities and further delaying the mobilisation of an emergency response. While in the case of doctors and international health workers, this paralysis was founded on biomedical knowledge of the virus and Ebola’s high case fatality rate, for the affected communities these fears seem to have been fuelled more by distrust of foreign medical teams and distant urban political elites.

A good illustration of the disconnect between these earlier and later constructions of Ebola were the briefings given by WHO officials in Freetown in March, just as Ebola was spreading more widely in the Guinea-forestière region and spilling over into Kailahun, in south-eastern Sierra Leone. Present at those briefings was Oliver Johnson, a medic and global health specialist from King’s College London who had arrived in Freetown a year earlier to establish a partnership with the Connaught Hospital. Mindful of the reports coming out of Guinea, Sierra Leone’s then health minister Miatta Kargbo convened an Ebola task force and invited WHO officials and representatives of MSF, the Red Cross, GOAL and UNICEF to participate. As a newcomer to the NGO sector with no experience of Ebola, Johnson was looking to more experienced hands for guidance, but the message he took home from both WHO and MSF officials was that there was nothing to fear because ‘there’s never been a major urban outbreak of Ebola, there’s no risk of this’. By late April, as the initial surge in cases in Guinea began to slow and WHO experts thought the epidemic had peaked, officials became even more complacent. ‘People were saying, “Oh it’s in Liberia and Guinea, but we’ve done such a good job, we’ve dodged a bullet,” Johnson recalled. “Maybe Sierra Leone won’t have too many cases”’.^57^ The complacency of officials in Sierra Leone contrasts with the urgent messages from WHO fieldworkers in Conakry who by mid-April were warning of spreading ‘panic and fear’ and urging Geneva that containment of the outbreak required a ‘drastic change of course’.^58^ These communications appear to have had little impact, however. On 17 April, 2014, Geneva upgraded the emergency to grade 2 and warned that the outbreak might require intensive surveillance and response activities for a further two to four months, but by mid-May the WHO was reporting that the outbreak was slowing, persuading several international NGOs, although not MSF, to redeploy their staff to other countries.^59^

As is now known, the WHO’s assessment was almost certainly due to misleading data. Official counts based on people presenting at treatment units with either confirmed or probable Ebola dipped in the middle of April to a new low, and even in May the number of new cases recorded in Guinea each week never exceeded 50. Liberia, which had reported 21 laboratory confirmed cases and 10 deaths in the first week of April, saw similar declines and by the end of May had reported no new cases since 9 April.^60^ However, in June there an increase in patients presenting with Ebola in Sierra Leone and Liberia, and by the final week of July aggregate cases in the three countries were running at in excess of 250 a week.^61^ What caused this dip followed by a marked resurgence is unclear but it would appear a key factor was distrust of foreign health workers, coupled with rumours about the goings on inside Ebola Treatment Units (ETUs).

As Fairhead and others have documented, such rumours were remarkably similar across the Ebola zone and commonly included accusations of blood stealing and claims that local politicians had conspired to infect people with Ebola as part of a plot to attract foreign aid funds.^62^ In an echo of the Reston incident and the original framing of Ebola as a potential biowarfare agent, there were even rumours that the virus had been manufactured in a laboratory by the US military.^63^ It is tempting to dismiss such rumours as hearsay or superstitions on a par with African beliefs in witchcraft and vampirism. However, as Luise White reminds us, rumours can also be read as ‘narratives, explanations, and theories in which colonial bureaucracies, corporations, events, and diseases are subjects’, and that rather than dismissing them as false or fantastical we might do better to regard them as products of local cultural conflicts and critiques of power.^64^ Graboyes makes a similar point when she argues that ‘public ignorance’ models of rumours fail to take account of Africans’ different understandings of risk and ‘of what constitutes the medical research encounter’. In particular, she argues that rumours about blood stealing reflect ‘the history of interactions with colonial government in the realm of health and disease’ and Africans’ experience of mass screening programmes, many of which have delivered little in the way of treatments or other tangible health benefits, as well as the negative regard with which biomedical researchers and government officials are generally held.^65^

A full exploration of the role of rumour in engendering social resistance to the Ebola response is beyond the scope of this paper. However, it is worth noting that in previous outbreaks in Africa, medical teams had worked hard to forestall the impact of such rumours by engaging with village headmen and other prominent community leaders. Pioneered by Jean-Jacques Muyembe-Tamfum, Director of the National Institute for Biomedical Research in Kinshasa, during the Kikwit outbreak, these community engagement methods proved highly effective in August 2014 when, in the midst of the West African outbreak, Ebola revisited the DRC. Muyembe, who was in Liberia at the time, immediately returned to Kinshasa and dispatched community relay teams to the site of the outbreak in Jeera County, Equateur Province, to facilitate safe but dignified burials in accordance with traditional customs. The result was that Muyembe was able to end the outbreak within three months with a total of just 66 cases.^66^

The importance of community engagement was well known to other Ebola experts, including David Heymann, who had worked alongside Muyembe at the Yambuku and Kikwit outbreaks, but somehow these lessons appear to have been overlooked in the crucial early weeks of the West African epidemic. Instead, the arrival of foreign medical teams in Guinea-forestière sparked violent clashes, especially in and around MSF installations in Macenta where government-imposed restrictions on traditional burials resulted in attacks on expatriate workers and in claims that Ebola did not exist or was being spread by outsiders.^67^ Similar resistance was encountered by Red Cross teams in other parts of the country.^68^

In its final report on the epidemic, the WHO Ebola Interim Assessment Panel expressed dismay at the absence of community engagement during the initial phase of the response, finding that ‘culturally sensitive messages and community engagement were not prioritized’.^69^ Muyembe, who sat on the assessment panel, believes that one reason for this oversight was that by 2014 officials in Geneva regarded Ebola ‘as a neglected disease of Central and Eastern African countries’ and expected the outbreak in Guinea to follow the same pattern as in other parts of the continent.^70^ Distrust of foreign medical teams was also undoubtedly exacerbated by the very different clinical outcomes observed for Western health workers, many of whom were airlifted to Europe and US for life-saving treatment with unlicensed drugs such as ZMapp that were not readily available to Africans. The irony is that ZMapp, like other experimental drugs and vaccines, only existed because it had been a priority for US biodefense. However, rather than conduct safety studies and begin phased trials of drugs and vaccines that showed experimental promise, after the initial laboratory research the products languished on the shelves of biotech companies.^71^

Perhaps because of its early experience on the ground in Guinea and its sensitivities to the ethical dilemmas presented by the limited availability of these medical products, MSF was one of the first to warn of the dangers presented by the distrust of foreign medical teams. Speaking at the Chatham House meeting in London in May, Armand Sprecher, an MSF emergency physician who had recently returned from a tour of duty in Conakry, warned that the international health community had ‘a marketing problem’.

Our best response to this marketing problem is to produce good advocates, survivors who can say and bear witness to what goes on inside the treatment units, to tell everyone that we do have their best interests at heart, that we are trying to save people. The problem is, in order to have survivors, you need patients. In order to get patients, you need survivors. Unfortunately, we’re caught in a catch 22.^72^

Ten months later, at a conference on Ebola at the Institute of Medicine in Washington DC, Sprecher returned to the theme. Recalling how at the same time as WHO officials were reporting a dip in cases in Conakry, MSF clinicians in Guéckédou in southern Guinea had witnessed a sudden spike in the case fatality rate, he explained:

All of a sudden we’re having patients who have to come in, they cannot hide their illness, they’re so obviously deathly ill that in their communities they cannot hide from us anymore…That’s not what the end of an outbreak looks like. The end of the outbreak is a lot of admissions and the number of confirmed cases is very low, it’s not having a handful of admissions who die very quickly on you. That was the clue that things were not going well.

Sprecher labelled this dip in cases observed in Conakry in April ‘the dog that didn’t bark’.^73^

The misleading data from Guinea was not only due to high levels of societal distrust, however. Another factor was Guinean authorities’ refusal to collect information on suspected Ebola cases and to paint what Keiji Fukuda would later characterise as an ‘overly rosy’ picture of the outbreak was painted.^74^ Indeed, in July, Pierre Formenty, a leading French haemorrhagic fever expert and head of the WHO’s Emerging and Dangerous Pathogens team, complained that the Guinean government was only announcing confirmed cases of Ebola so as to ‘ “minimize” artificially the magnitude of the Ebola outbreak to reassure expatriates working in the mining industry’.^75^ By now MSF was warning that the outbreak was ‘totally out of control’ and that it was in a ‘race against time’ to stop the epidemic, prompting WHO officials to accuse MSF of exaggerating the risk and fomenting ‘unnecessary panic’.^76^ But MSF was not exaggerating: the virus had most likely crossed into Liberia and Sierra Leone in late April or early May from where it spread via several concurrent transmission lines, aided by the passage of migrant workers, the majority of whom belonged to the same Kissy ethnic group, across the porous borders of the Manu River region.

The first person in whom the infection was recognised in Sierra Leone was a pregnant woman who had presented with a high fever at Kenema General Hospital on 24 May, miscarrying soon afterwards.^77^ Like other patients who were diagnosed with Ebola in Kenema over the following weeks, the woman had recently attended the funeral of a traditional healer from Koindu, who had died on 30 April after treating several people who had crossed the Guinean border to seek her assistance.^78^ Indeed, on the same day that the pregnant woman tested positive for Ebola so did blood from another desperately ill woman in Kailahun, as well as that of a third woman who had also visited the healer. Retrospective genetic analysis of virus samples taken from 78 Ebola patients in Sierra Leone between May and mid-June subsequently showed that at least 14 had been infected at the healer’s funeral, giving rise to two distinct lineages of the virus.^79^ Tragically, one lineage was traced to a nurse who, instead of seeking treatment at a clinic in Kailahun, where staff had been trained to watch out for Ebola patients, had bypassed the hospital and driven through the night to Daru, her home village. In the process, she infected a truck driver and four other people, setting off new transmission chains all over Sierra Leone.^80^ The result was that there was a delay of at least four weeks between Ebola entering Sierra Leone and recognition of the first cases, a period in which infectious contacts were able to diffuse the virus throughout Kailahun and Kenema, challenging Sierra Leone’s capacity to contain the outbreak on its eastern borders. Unfortunately, it would appear that one or both of these Sierra Leone lineage viruses were subsequently reintroduced to Guinea, reigniting the epidemic there just as the original virus was dying out.^81^

There were also other factors that contributed to the rapid diffusion of Ebola in Sierra Leone, not least the absence of doctors and health workers trained in Ebola containment. However, one factor that stands out in view of the gaps identified ten years earlier by Bausch and Rollin was the absence of laboratory diagnostics. Indeed, at the time of the outbreak the only laboratory in the tri-border region capable of tracking the movement of Ebola was the VHF laboratory at Kenema General Hospital – a facility that had been set up to monitor Lassa fever with the support of Tulane University and funding from the US National Institutes of Health.^82^ When it became clear that Kenema had a major outbreak on its hands, this became the go-to laboratory for diagnostic assays using real-time reverse transcription polymerase chain reaction (RT-PCR) and by early June the Kenema facility had been repurposed by the Ministry of Health and Sanitation to take the lead in contact tracing. However, the team consisted of just two people, one to run diagnostics and another to conduct follow-up surveillance, and although the WHO sent a contract epidemiologist from GOARN to assist, by mid-June the diagnostics group was overwhelmed. ‘We had two and half people to monitor 300 contacts in Kenema every day,’ recalled Nadia Wauquier, the Tulane researcher in charge of laboratory assays for the Lassa Fever Research Programme at Kenema. ‘So that was impossible. It felt like we were under siege’.^83^

**Figure 2:**
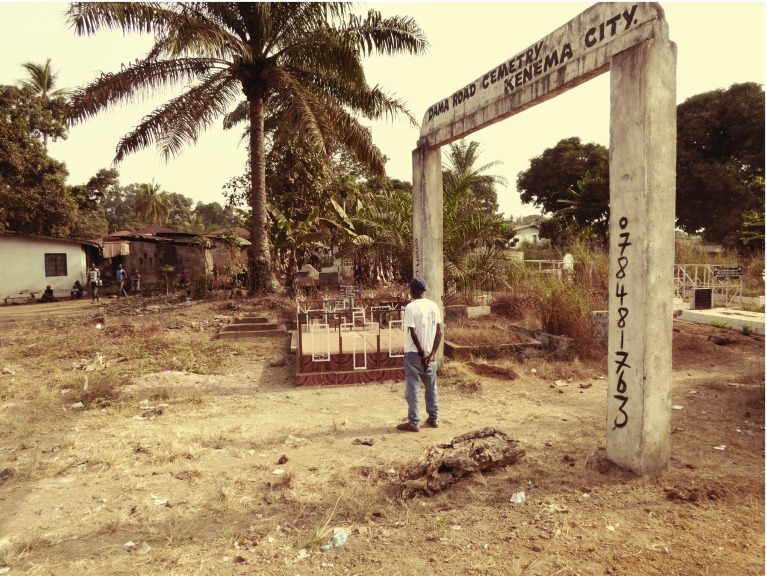
Mohammed Sow, a driver for the Tulane Lassa Fever Research Programme, visits the grave of Mbalu J. Fonnie at Dawa Road cemetery, Kenema. The matron of Kenema General Hospital, Fonnie was one of 11 nurses who lost their lives when Ebola ripped through wards in July–August 2014. The outbreak also claimed the life of Dr Sheik Humarr Khan, the hospital’s chief physician and Sierra Leone’s leading Lassa specialist. Source and credit: Mark Honigsbaum

This sense of siege was exacerbated by the government’s decision to refer all suspected Ebola cases from Freetown to Kenema – a gruelling five-hour journey by road in overheated ambulances. On one level, this policy made sense: Kenema was the only place capable of carrying out PCR in Sierra Leone and one of the few hospitals in the country with staff trained and equipped to treat VHFs. However, Kenema was also a stronghold of the opposition Sierra Leone People’s Party and during the civil war it and Kailahun had been centres of rebel activity. The result was that when ambulances carrying Ebola patients began arriving in Kenema, rumours began spreading that the epidemic was a plot by the ruling All People’s Congress party and that nurses on the wards were deliberately infecting patients with Ebola in order to attract foreign aid for the benefit of the political elite in Freetown.^84^ Dr Sheik Humarr Khan, the hospital’s chief physician and the country’s leading Lassa specialist, who managed the Lassa Fever Research Programme with Tulane, attempted to counteract these rumours by travelling to the epicentre of the outbreak in Kailahun to hold a series of educational workshops. But villagers resisted his entreaties to evacuate suspect cases to Kenema for testing. Opposition was particularly fierce in the healer’s home town of Koindu where the population erected roadblocks and broke the windshield of Khan’s government-issued Toyota. ‘There were rumours that we were coming to give them disease,’ said Robert Garry, a VHF specialist from Tulane who had flown to Kenema to assist Khan. ‘They said we would take people away and never come  back’.^85^ Tragically, it would take the death from Ebola of several Kenema nurses and laboratory staff, followed, at the end of July, by the death of Khan himself, to convince WHO officials in Freetown that this was unlike previous Ebola outbreaks and required exceptional measures (see Figure 2).^86^

In Liberia health facilities were also completely unprepared for the arrival of Ebola from Guinea and medics had to learn the lessons of previous outbreaks all over again. A key factor was local physicians’ unfamiliarity with Ebola and a lack of understanding of the disease’s diverse symptomatology. A good example came at Foya Borma hospital in Lofa district, the site of what is widely believed to have been Liberia’s index case. CDC epidemiologists traced the introduction of Ebola there to a woman who had arrived from Guéckédou in April. At the time Liberia had no laboratory capable of identifying Ebola and because the woman presented with severe diarrhoea, the attending physician assumed she had cholera. Even when on day two she began to display haemorrhagic symptoms, he did not consider Ebola, reasoning that she must be co-infected with Lassa fever.^87^ This is a case where regard for the haemorrhagic symptoms so vividly described by Preston would have been useful; but, even if the physician had had a copy of *The Hot Zone* to hand he may still not have recognised the woman’s symptoms as, despite the close genetic relationship between the Guinean and Zairean stains of EBOV, in infections caused by the Guinean strain the most prominent features are diarrhoea and hiccups, haemorrhaging being secondary.^88^

By May the CDC had contact tracing teams in place in Foya and by June both it and the WHO were confident that Liberia was free of Ebola, having gone through two full incubation periods (21 days times two) without any new cases being registered. Unfortunately, these assessments were wrong: retrospective phylogenetic analysis suggests that there were now at least three related strains of the virus circulating simultaneously in West Africa.^89^ In mid-July one or more of these strains was responsible for a new surge of cases in Liberia and by mid-August Ebola had arrived in the capital Monrovia. The pictures of desperately ill patients lying abandoned on the streets of the Liberian capital for want of spaces inside ETUs appears to have been a game-changer, shocking people out of their lethargy and persuading communities to take on board the biomedical messaging. According to Kevin De Cock, the head of CDC’s mission to Liberia, the critical period was in August when there were heavy rains and bodies that had been hurriedly buried began floating to the surface, sparking public outrage and the imposition of mandatory cremations.^90^ At the same time, De Cock observed, people spontaneously stopped touching each other and travelled less, ‘so that in certain cases the communities implement[ed] their own quarantine and isolation’.^91^

Soon after, similar behavioural shifts also occurred spontaneously in Sierra Leone, particularly in Kailahun and Kenema. In Bo, Bombali and Tonkolili the authorities also achieved high levels of community compliance by engaging with paramount chiefs and district heads. However, in the Western District and Port Loko community engagement was less successful and there were several cases of village headmen deliberately flouting the Ebola control regulations by concealing cases or failing to report burials. The reason for this is in unclear but a likely factor was the insistence by Sierra Leonean military and government officials on quarantines and other harsh control measures long after they had been shown to be counter-productive.^92^

However, for all that the increasingly visible deaths from Ebola persuaded communities in the affected countries to take the threat more seriously, the AP emails suggest that WHO officials were still reluctant to treat the epidemic as anything more than a regional health crisis, albeit a severe one. The event that arguably changed this was the arrival at Lagos airport on 20 July of a visibly ill Liberian-American lawyer named Patrick Sawyer. The episode is instructive not only because of the way it focused attention on the wider security threat posed by the outbreak – with a population of 1.4 million, Lagos is one of the populous cities in Africa and its airport a gateway to several international destinations, including the United States – but the light it shone on the relative neglect of core health capacities in Liberia compared to other countries in central and southern Africa. In particular, it is argued, Nigeria, like other African countries that were badly affected by the HIV/AIDs pandemic, had benefited from resources provided by the President’s Emergency Plan for AIDS Relief (PEPFAR) and the Global Fund to Fight AIDS, Tuberculosis and Malaria (GFATM). By contrast, Liberia, Sierra Leone and Guinea had escaped the worst of the AIDs pandemic but also missed out on many of the HIV-related investments that have had positive health benefits for the control of other diseases.^93^ Indeed, when Sawyer fell sick he reportedly used his influence with Liberian finance ministry officials to board a flight to Lagos calculating he would receive better treatment in the Nigerian capital. Unfortunately, on arrival at the airport, Sawyer collapsed and was rushed to the First Consultant Hospital, dying five days later. In the process, he infected 19 people, including the Nigerian physician who treated him.^94^ In the end it was only thanks to strict hygiene controls and rigorous contact tracing that a wider outbreak was averted.^95^

The incident raised the prospect that Ebola could be in any city on the globe in a matter of hours.^96^ As De Cock put it, the Sawyer case ‘was everybody’s worst nightmare’, exactly the sort of scenario that had been envisaged by the IoM report in the early 1990s when it warned about the dangers that the growth in international travel and commerce posed for the global spread of EIDS.^97^ It was around this time that Nancy Writebol and Kent Brantly, two American missionaries who had been caring for patients at a treatment centre in Monrovia, also fell ill. When on 1 August they were airlifted to Georgia for emergency treatment at Emory Hospital, Atlanta, the news sparked widespread coverage in the US media and furious tweets from the presumptive Republican presidential candidate Donald Trump and other right-wing pundits.^98^ At a summit meeting in Guinea the same day Margaret Chan announced a $100-million plan to combat the Ebola epidemic, admitting ‘this outbreak is moving faster than our efforts to control it’.^99^ Six days later, on 8 August, Chan finally bowed to pressure from MSF and the CDC and declared a pheic.

De Cock, a former director of the WHO’s Department of HIV/AIDS and an expert on neglected tropical diseases with more than thirty years experience in the field, believes it was the evacuation of the American missionaries to Emory Hospital, coupled with the presentation in late September of a Liberian national infected with Ebola at Dallas Presbyterian Hospital, Texas, that finally shifted global perceptions of Ebola as ‘some kind of obscure virus that you read about from time to time…[to being] an issue for global health security’.^100^

## 4 Conclusion

On 19 September 2014, in recognition of the ‘international threat to peace and security’ posed by the outbreak in West Africa, the UN Secretary General established the United Nations Mission for Ebola Emergency Response (UNMEER) to scale up the emergency response and co-ordinate the delivery of logistical, technical and financial support to the Ebola zone.^101^ It was only the second time in history that an infectious disease outbreak had been debated on the floor of the UN – the first time had been AIDS in 1987– and it had a similarly galvanising effect. In mid-September President Obama pledged to send 3000 troops to Liberia, and by the end of the year the US Congress had agreed emergency funding of $5.4 billion for Ebola, more than had previously been allocated for any EID.

Just as in the 1990s, when the publication of the IoM report and the growing concern about the security threat posed by Ebola and other emerging infections, proved highly effective at focusing the attention of politicians, so it proved again. The result was that with the dispatch of thousands of health professionals to West Africa in late 2014 to staff newly constructed ETUs, and with the establishment of dozens of diagnostic laboratories and the fast-tracking of trials of unlicensed medical products, by 2015 Ebola was no longer in danger of being neglected. But that only begged the question how the WHO and other experts had overlooked the dangers posed by the outbreak in Guinea-forestière in the first place? In the aftermath of the crisis, there was no shortage of committees and panels willing to answer that question.

One of the most damning verdicts came from Dame Barbara Stocking, the chair of the WHO’s Ebola Interim Assessment Panel. Expressing bafflement as to why ‘early warnings…did not result in an effective and adequate response’, Stocking concluded that what had been lacking was ‘independent and courageous decision-making by the Director-General and the WHO Secretariat’. Stocking recommended that to avoid such mistakes in future the WHO should consider an intermediate level of alert to draw the attention of the global health community to events falling between routine public health risks and those that might require the declaration of a pheic.^102^

Harvard’s Global Health Institute and the London School of Hygiene and Tropical Medicine (LHSTM) was similarly critical of senior officials in Geneva. However, rather than laying the failures at the door of a single individual, the panel concluded that the slow response reflected a lack of clarity about a range of governance issues across the WHO, as well as the roles and responsibilities of member countries, and proposed ‘ten essential reforms’ to the organisation.^103^ Meanwhile, other groups identified ‘gaping holes in preparedness’ and used the inquests to call for the establishment of a $1 billion Pandemic Product Development Committee to scale up research and development of drugs and vaccines for EIDs – a strategic move Paul Farmer would surely appreciate.^104^

This paper has offered a different diagnosis. Rather than focusing on the relationship between officials in Geneva and AFRO and the structure of the WHO, I have suggested it would be more profitable to examine the discursive and non-discursive practices that produced Ebola as a ‘problem’ for global health security. Answering that question, has led to a consideration of the history of Ebola and the shifting medical and security constructions of the virus. Central to those constructions was Ebola’s framing as an iconic EID and potential biowarfare agent in the early 1990s – constructions that can be traced back to the Reston incident, the IoM report, and the popularity of books like *The Hot Zone*. However, while these constructions of Ebola were highly effective at unlocking research dollars and mobilising political attention, they also obscured other constructions of the virus and ways of responding to Ebola outbreaks. The result was that as haemorrhagic fever experts became familiar with the clinical management and containment of Ebola, and as Ebola began to be configured as a rare disease of remote forested regions of central Africa, so the virus slipped down the ranking of global health and international security concerns.

This relegation of Ebola to the fringes of global health security is nowhere better illustrated than by the position it occupied in the decision instrument for pheics in the IHR (2005). Unlike outbreaks of smallpox, polio or new influenza subtypes, which required automatic notification to the WHO, Ebola was subjected to a set of algorithms and triage mechanisms that enabled officials to judge when and at what point and an outbreak might pose a ‘significant risk of international spread’ and might require a higher grade alert and, possibly, the declaration of a pheic. By applying managerial procedures contained in the ERF to these determinations, the aim was to introduce transparency into the deliberations of expert advisors, thereby avoiding the accusations of conflicts of interest that had greeted the WHO’s declaration of a pheic during the 2009 swine flu outbreak. At the same time, the IHR (2005) sought to encourage member states to strengthen core health capacities and take greater responsibility for the regulation of EID risks originating within their borders. The net result was to shift the burden for biosurveillance onto states that were ill-equipped to make such judgements and to de-emphasise the threat posed by Ebola at precisely the moment when the virus was crossing international borders for the first time.

Nevertheless, as shown by Chan’s rapid declaration of a pheic in relation to H1N1 swine flu in 2009 and polio in 2014, senior WHO officials could have chosen to override these bureaucratic procedures. That they chose not to is testimony to the extent to which by 2014 Ebola had become an object of medical and political neglect. Unlike polio, Ebola did not threaten to undermine long-standing WHO programmes and investments in disease eradication. Nor were there vaccines and drugs ready for deployment to the Ebola zone. On the contrary, research into promising investigational Ebola products had ceased to be a priority.

Even if Ebola medications had been available, WHO officials, like other experts, had little reason to believe that Ebola posed a wider epidemic threat to the region. This was because in the past the rapid isolation of infectious patients and the speedy burial of victims, coupled with contact tracing and other control measures, had greatly foreshortened transmission chains. And even where, as in Kikwit in 1995, the virus had been circulating in the community for thirteen weeks before the deaths of health workers brought it to the attention of international experts, once GOARN had been activated the outbreak was quickly brought under control. In short, as WHO officials informed Oliver Johnson in Freetown in March 2014, ‘Ebola doesn’t cause urban outbreaks’. But, of course, Ebola had never been observed as far west as Guinea before. Nor had previous outbreaks occurred close to the borders of three countries or in a region where protracted civil wars had left weakened and fragmented health systems and a legacy of distrust between rural populations and urban political elites. Nor had previous outbreaks visited border regions with such mobile populations or where new road and transport systems had greatly reduced travel times between villages and cities.

Ebola’s shifting medical identity also helps explain why, once it became clear that Ebola was spreading faster and more widely than anticipated, the WHO and other agencies appeared to become paralysed by fear, further delaying the deployment of responders to West Africa. This paralysis is best understood as a result of the misleading early media reports from Guinea and Liberia – reports that played on the popular image of Ebola as a highly infectious hot virus with the ability to cause severe haemorrhaging and other terrifying clinical symptoms. By contrast, communities in the affected countries did not buy into this image of Ebola; instead, their fears were driven by rumours about the ‘real’ motivations of foreign medical teams and distrust of ETUs. Social resistance was further aggravated by bans on traditional burials and the failure to adopt community engagement methods that had proved so effective in winning the trust of rural populations during Ebola outbreaks in other parts of Africa – evidence of yet another aspect of the neglect of knowledge of Ebola. The net result was to depress the true level of Ebola infections in Guinea and persuade WHO officials that there was no need for further deployment within GOARN.

Following Stocking’s call for a new intermediate level of alert, the WHO’s IHR Review committee has proposed a new category of risk called an International Public Health Alert.^105^ However, my reading of the WHO’s faltering response to the 2013–16 outbreak suggests that tinkering with the managerial procedures for triaging between Ebola and other EID risks is unlikely to resolve these biosecurity problematisations. On the contrary, like the ‘false alarm’ over swine flu in 2009, such ‘missed alarms’ may be an inescapable consequence of pandemic preparedness systems that seek to rationalise responses to novel emergence events.

## Footnotes

1.
‘Afro-memorandum, 25 March 2014’, in ‘Bungling Ebola-Documents’, Associated Press, http://data.ap.org/projects/2015/who-ebola/(accessed 17 June 2015); Sylvain Baize *et al.*, ‘Emergence of Zaire Ebola Virus Disease in Guinea’, *New England Journal of Medicine*, 371, 15 (2014), 1418–25.10.1056/NEJMoa140450524738640

2.
‘WHO, Emergency Response Framework (ERF)’, http://www.who.int/hac/about/erf/en/(accessed 17 June 2015).

3.
‘WHO, International Health Regulations (2005)’, Annex 2, 43, http://www.who.int/ihr/publications/9789241596664/en/(accessed 17 June 2015).

4.
‘Briefing note to the director-general, June 2014’, Associated Press, *op. cit.* (note 1).

5.
‘A last resort, 4 June 2014’, Associated Press, *op. cit.* (note 1).

6.
‘Ebola Outbreak in W. Africa “Totally Out of Control” – MSF’, *RT English*, http://www.rt.com/news/167404-ebola-africa-out-of-control/(accessed 30 September 2015).

7.
‘Ebola: Pushed to the Limit and beyond’, Médecins Sans Frontières International, http://www.msf.org/article/ebola-pushed-limit-and-beyond(accessed 29 April 2015); see also Lawrence O. Gostin and Eric A. Friedman, ‘A Retrospective and Prospective Analysis of the West African Ebola Virus Disease Epidemic: Robust National Health Systems at the Foundation and an Empowered WHO at the Apex’, *The Lancet*, 385, 9980 (2015) 1902–9; ‘WHO, Report of the Ebola Interim Assessment Panel – July 2015’, http://www.who.int/csr/resources/publications/ebola/ebola-panel-report/en/ (accessed 6 August 2015).10.1016/S0140-6736(15)60644-425987158

8.
Adam Kamradt-Scott, ‘WHO’s to Blame? The World Health Organization and the 2014 Ebola Outbreak in West Africa’, *Third World Quarterly*, 37, 3 (2016), 401–18; Colin McInnes, ‘WHO’s Next? Changing Authority in Global Health Governance after Ebola’, *International Affairs*, 91, 6 (2015), 1299–1316.10.1111/1468-2346.12454PMC710857732287367

9.
Stephen J. Collier, Andrew Lakoff and Paul Rabinow, ‘Biosecurity: Towards an Anthropology of the Contemporary’, *Anthropology Today*, 20, 5 (2004) 3–7.

10.
Melinda Cooper, ‘Pre-Empting Emergence: The Biological Turn in the War on Terror’, *Theory, Culture and Society*, 23 (2006), 113–35; Jean Comaroff, ‘Beyond Bare Life: AIDS, (Bio)politics, and the Neoliberal Order’, *Public Culture*, 19 (2007), 197–219; Frédéric Keck, *Un Monde Grippé* (Paris: Flammarion, 2010); Theresa MacPhail, ‘A Predictable Unpredictability: The 2009 H1N1 Pandemic and the Concept of “Strategic Uncertainty” Within Global Public Health’, *Behemoth A Journal of Civilisation*, 3, 3 (2010), 57–77; Sudeepa Abeysinghe, ‘Zika, Uncertainty and Global Health Policy’, *Somatosphere*, 17 February (2016).

11.
The interviews were conducted in Sierra Leone, London, Washington DC and Atlanta between March 2015 and February 2016 as part of an oral history project funded by the Wellcome Trust and included interviews with government ministers, health responders, clinical researchers, nurses, Ebola survivors, members of NGOs and key international scientific experts and agency officials (grant number 107721/Z/15/Z).

12.
, While ‘Ebola virus’ was the name given to the virus isolated in 1976 in the first outbreak in Yambuku in the DRC (former Zaire), in 2002 the species name was contracted to *Zaire ebolavirus*or ZEBOV for short. However, as many writers continued to use the generic term to describe both the Zairean and other subtypes, in 2010 it was proposed to retain the previous species names but to revert to the common name used in the field, with the Zaire strain being known as EBOV. Jens H. Kuhn *et al.*, ‘Proposal for a Revised Taxonomy of the Family Filoviridae: Classification, Names of Taxa and Viruses, and Virus Abbreviations’, *Archives of Virology,* 155, 12 (2010), 2083–2103.10.1007/s00705-010-0814-xPMC307419221046175

13.
Baize, *op. cit.* (note 1).

14.

Paul Farmer , ‘Social Inequalities and Emerging Infectious Diseases’, Emerging Infectious Diseases, 2, 4 (1996), 259–269.896924310.3201/eid0204.960402PMC2639930

15.

João Nunes , ‘Ebola and the Production of Neglect in Global Health’, Third World Quarterly, 37, 3 (2016), 542–556.

16.

Jean-Jacques Muyembe-Tamfum , ‘Ebola Virus Outbreaks in Africa: Past and Present’, Onderstepoort Journal of Veterinary Research, 72, 2 (2012), 451; WHO, ‘Table: Chronology of Previous Ebola Virus Disease Outbreaks’, http://www.who.int/mediacentre/factsheets/fs103/en/ (accessed 12 November 2015).10.4102/ojvr.v79i2.45123327370

17.
‘Ebola and Emerging Infectious Diseases: Measuring the Risk’, Royal Institute of International Affairs, Chatham House, 6 May 2014, https://www.chathamhouse.org/events/view/198881(accessed 12 November 2015).

18.
Pierre Rollin, interview with author, Centers for Disease Control and Prevention, Atlanta, 26 October 2015.

19.

J. Knobloch , E. J. Albiez and H. Schmitz , ‘A Serological Survey on Viral Haemorrhagic Fevers in Liberia’, Annales de l’Institut Pasteur/Virologie, 133, 2 (1982), 125–128.

20.
The Ebola virus was isolated by scientists at the CDC and Porton Down from blood samples forwarded from Yambuku via the Institute of Tropical Medicine in Antwerp. The first filovirus to be recognised was Marburg in 1967. Karl M. Johnson *et al.*, ‘Isolation and Partial Characterisation of a New Virus Causing Acute Hæmorrhagic Fever in Zaire’, *The Lancet*, 309, 8011 (1977), 569–71.10.1016/s0140-6736(77)92000-165661

21.

Richard Preston , The Hot Zone (London and New York: Doubleday, 1994), 81–83.

22.

Nicholas B. King , ‘Security, Disease, Commerce Ideologies of Postcolonial Global Health’, Social Studies of Science, 32, 5–6 (2002), 763–789.

23.

Laurie Garrett , The Coming Plague: Newly Emerging Diseases in a World Out of Balance (New York: Farrar, Straus and Giroux, 1994), 593–595. The war game exercise uncannily presaged the 2013–16 Ebola epidemic, imagining an outbreak at the border of three fictitious equatorial countries where civil war had led to a dangerous concentration of refugees living in unsanitary encampments.

24.
David Heymann, interview with author, London, 19 March 2015.

25.
Joshua Lederberg, Robert E. Shope and S. C. Oaks (eds), *Emerging Infections: Microbial Threats to Health in the United States* (Washington DC: National Academy Press, 1992), 34–5, 83.25121245

26.
Stephen S. Morse (ed.), *Emerging Viruses* (New York: Oxford University Press, 1993); Garrett, *op. cit.* (note 23); Richard M. Krause (ed.), *Emerging Infections*, Biomedical Research Reports (San Diego, CA: Academic Press, 1998).

27.
Lederberg, Shope and Oaks, *op. cit.* (note 25), 42, 47.

28.
See Nicholas G. Evans, Tara C. Smith and Maimuna S. Majumder (eds), *Ebola’s Message: Public Health and Medicine in the Twenty-First Century* (Cambridge, MA, and London: The MIT Press, 2016); Daniel G. Bausch and Lara Schwarz, ‘Outbreak of Ebola Virus Disease in Guinea: Where Ecology Meets Economy’, *PLoS Neglected Tropical Diseases*, 8, 7 (2014), e3056.10.1371/journal.pntd.0003056PMC411759825079231

29.

C. J. Peters and J. W. Peters , ‘An Introduction to Ebola: The Virus and the Disease’, Journal of Infectious Diseases, 179, 1 (1999), ix–xvi.10.1086/5143229988154

30.

David Quammen , Ebola: The Natural and Human History (London: Bodley Head, 2014), 31.

31.
‘Ebola Vaccine Research and NIH Funding Cuts: Reviewing the Numbers’, *PolitiFact*, http://www.politifact.com/truth-o-meter/article/2014/oct/17/was-ebola-vaccine-research-hindered-nih-funding-cu/(accessed 14 June 2015).

32.
Gostin and Friedman, *op. cit.* (note 7).

33.
King, *op. cit.* (note 22); Theodore M. Brown, Marcus Cueto and Elizabeth Fee, ‘The World Health Organization and the Transition from “International” to “Global” Public Health’, *American Journal of Public Health*, 96 (2006), 62–72; Lorna Weir and Eric Mykhalovsky, ‘The geopolitics of global public health surveillance in the twenty-first century’, in Alison Bashford (ed.), *Medicine At The Border* (Basingstoke: Palgrave Macmillan, 2006), 240–64.10.2105/AJPH.2004.050831PMC147043416322464

34.
Launched by the WHO in 2000, GOARN is a virtual network drawing on 120 institutional partners worldwide that links institutions and individuals with specialist knowledge of and expertise in epidemics. Its first test came in March 2003 when the WHO issued an alert about a new respiratory virus in Hong Kong – what would later be identified as SARS. Within four months, GOARN had traced all the chains of transmission and interrupted an outbreak that affected 27 countries worldwide. David Heymann and Rodier Guénaël, ‘Global Surveillance, National Surveillance, and SARS’, *Emerging Infectious Diseases*, 10, 2 (2004), 173–5.10.3201/eid1002.031038PMC332293815040346

35.

Daniel G. Bausch and Pierre E. Rollin , ‘Responding to epidemics of ebola hemorrhagic fever: progress and lessons learned from recent outbreaks in Uganda, Gabon, and Congo’, in Barbara E. Murray , W. Michael Scheld and James M. Hughes (eds), Emerging Infections 6 (Washington DC: American Society of Microbiology, 2004), 35–58.

36.
Frederick A. Murphy and C. J. Peters, ‘Ebola virus: where does it come from and where is it going?’ in Richard M. Krause (ed.), *Emerging Infections*, Biomedical Research Reports (San Diego, CA: Academic Press, 1998), 375–410.

37.
Bausch and Rollin, *op. cit.* (note 35).

38.
Muyembe-Tamfum *et al*, *op. cit.* (note 16).

39.
WHO, ‘Ebola Virus Disease, Fact Sheet 103, Updated August 2015. Table: Chronology of Previous Ebola Virus Disease Outbreaks’, http://www.who.int/mediacentre/factsheets/fs103/en/(accessed 4 December 2015).

40.
Bausch and Rollin, *op. cit.* (note 35).

41.
David Heymann and Rodier Guénaël, *SARS: Lessons From A New Disease* (National Academies Press, 2004), https://www.ncbi.nlm.nih.gov/books/NBK92444/ (accessed 30 November 2016).

42.
Pierre Formenty, ‘WHO Ebola Diaries: First Signals – March 2014’, http://www.who.int/features/2015/ebola-diaries-formenty/en/ (accessed 28 April, 2015); Laurie Garrett, ‘Ebola’s Lessons’, *Foreign Affairs*, https://www.foreignaffairs.com/articles/west-africa/2015-08-18/ebolas-lessons (accessed 3 November 2015).

43.
Kamradt-Scott, *op. cit.* (note 8).

44.
‘Implementation of the International Health Regulations (2005). Report of the Review Committee on the Role of the International Health Regulations (2005) in the Ebola Outbreak and Response’, WHO, 13 May (2016), 39.

45.
Heymann, *op. cit.* (note 24).

46.
For further discussion, see Mark J. Siedner *et al*, ‘Strengthening the Detection of and Early Response to Public Health Emergencies: Lessons from the West African Ebola Epidemic’, *PLOS Medicine*12, 3 (2015), 1–8.10.1371/journal.pmed.1001804PMC437188725803303

47.
Andrew Lakoff, ‘Two States of Emergency: Ebola 2014’, *Limn*, 5 (2015), http://limn.it/issue/05/(accessed 29 May 2015).

48.
The IHR (2005) defines a pheic as an ‘extraordinary event which is determined to constitute a public health risk to other States through the international spread of disease and to potentially require a coordinated international response’. WHO (2005), *op. cit.* (note 3).

49.
Stefan Elbe, *Security and Global Health* (Cambridge: Polity Press, 2010), 172–5.

50.

Nick Bingham and Steve Hinchcliffe , ‘Mapping the multiplicities of biosecurity’, in Andrew Lakoff and Stephen J. Collier (eds), Biosecurity Interventions: Global Health and Security in Question (New York: Colombia University Press), 173–191: 177.

51.
D. Cohen and P. Carter, ‘Conflicts of Interest: WHO and the Pandemic Flu “Conspiracies” ’, *BMJ*, 340 (2010), c2912; Sudeepa Abeysinghe, ‘An Uncertain Risk: The World Health Organization’s Account of H1N1’, *Science in Context*, 27, 3 (2014), 512–529.

52.
These obligations included monitoring bird flu in China, the MERS virus in Saudi Arabia, wild polio viruses in Africa, Asia and Syria, plus humanitarian crises such as Typhoon Haiyan in the Philippines in 2013.

53.
WHO (2005), *op. cit.* (note 3).

54.
‘WHO Statement on the Meeting of the International Health Regulations Emergency Committee Concerning the International Spread of Wild Poliovirus’, http://www.who.int/mediacentre/news/statements/2014/polio-20140505/en/(accessed 1 December 2015).

55.
By contrast, once the WHO had triggered a pheic, it played a key role in removing regulatory and financial hurdles to enable fast-track trials of experimental Ebola drugs and vaccines, creating ‘an exceptional political space’ for pharmaceutical development. Anne Roemer-Mahler and Stefan Elbe, ‘The Race for Ebola Drugs: Pharmaceuticals, Security and Global Health Governance’, *Third World Quarterly*, 37, 3, 487–506.

56.
At the time of the outbreak, the only laboratory in West Africa with the equipment to conduct rapid diagnostic tests for Ebola was the former Lassa fever laboratory at Kenema General Hospital. The next nearest laboratories were in Gabon and South Africa: Nadia Wauquier *et al.*, ‘Understanding the Emergence of Ebola Virus Disease in Sierra Leone: Stalking the Virus in the Threatening Wake of Emergence’, *PLOS Currents Outbreaks*, 20 April (2015), edition 1; Bausch and Rollin, *op. cit.* (note 35).10.1371/currents.outbreaks.9a6530ab7bb9096b34143230ab01cdefPMC442392525969797

57.
Oliver Johnson, interview with author, Freetown, 10 March 2015. In fact, it was untrue that there had never been an urban outbreak of Ebola: in 1995 the virus had triggered just such an emergency in Kikwit, a city in the Democratic Republic of the Congo (DRC) with 400 000 inhabitants. Because of weakened infectious disease surveillance systems, the initial cases were wrongly diagnosed as gastrointestinal disease, permitting the virus to spread unnoticed in the community for 13 weeks. It was only when health workers in Kikwit also became infected that the outbreak came to the attention of the authorities in Kinshasa and the WHO was alerted. David Heymann *et al*, ‘Ebola Hemorrhagic Fever: Lessons from Kikwit, Democratic Republic of the Congo’, *Journal of Infectious Diseases*, 179, 1 (1999), S283–86.

58.
‘We Need Support’, 14 April 2014. Associated Press, *op. cit.* (note 1).

59.
‘WHO, Ebola situation report, 13 May 2015’, http://www.who.int/csr/disease/ebola/situation-reports/archive/en/(accessed 17 June 2015).

60.
‘WHO, Global Alert and Response, One Year into the Ebola Epidemic: A Deadly, Tenacious and Unforgiving Virus’, WHO, 2015, http://www.who.int/csr/disease/ebola/one-year-report/introduction/en/(accessed 19 December 2016).

61.

Sylvie Briand , ‘The International Ebola Emergency’, New England Journal of Medicine, 371, 13 (2014), 1180–1183.2514085510.1056/NEJMp1409858

62.
James Fairhead, ‘Understanding Social Resistance to Ebola Response in Guinea, Ebola Response Anthropology Platform’, April 2015, http://www.ebola-anthropology.net/evidence/1269/(accessed 27 January, 2016); Samuel Cohn and Ruth Kutalek, ‘Historical Parallels, Ebola Virus Disease and Cholera: Understanding Community Distrust and Social Violence with Epidemics’, *PLoS Currents*, 26 January (2016), edition 1.10.1371/currents.outbreaks.aa1f2b60e8d43939b43fbd93e1a63a94PMC473943826865987

63.
Alan Feuer, ‘The Ebola Conspiracy Theories’, *The New York Times*, 18 October (2014).

64.

Luise White , Speaking With Vampires: Rumor and History in Colonial Africa (Berkeley, CA: University of California Press, 2008), 82, 312.

65.

Melissa Graboyes , The Experiment Must Continue: Medical Research and Ethics in East Africa, 1940–2014 (Athens, OH: Ohio University Press, 2015), 34, 46–7, 133.

66.
Mark Honigsbaum, ‘Jean-Jacques Muyembe-Tamfum: Africa’s Veteran Ebola Hunter’, *The Lancet*, 385, 9986 (2015), 2455; ‘WHO Declares End of Ebola Outbreak in the Democratic Republic of Congo’, 2014, http://www.who.int/mediacentre/news/statements/2014/drc-ends-ebola/en/(accessed 28 November 2016).10.1016/S0140-6736(15)61128-X26122060

67.
Fairhead; Cohn and Kutalek, *op. cit.* (note 62).

68.
‘Red Cross Ebola Teams in Guinea Attacked 10 Times a Month’, *Reuters*, 12 February (2015), http://www.reuters.com/article/us-health-ebola-guinea-idUSKBN0LG1GO20150212(accessed 29 November 2016).

69.
‘Report of WHO Interim Assessment Panel’, *op. cit*. (note 7), 20.

70.
Muyembe-Tamfum, email to author, 17 June 2015.

71.
Denise Grady, ‘Ebola Vaccine, Ready for Test, Sat on the Shelf’, *The New York Times*, 23 October (2014).

72.
Transcript, ‘Ebola and Emerging Infectious Diseases: Measuring the Risk’, Chatham House, 6 May 2014,https://www.chathamhouse.org/events/view/198881(accessed 11 November 2015).

73.
Armand Sprecher, ‘The MSF Response to the West African Ebola Outbreak’, The Ebola Epidemic in West Africa, *Institute of Medicine, Forum on Microbial Threats*, Washington DC, 25 March 2015.

74.
Keiji Fukuda, ‘Ebola in West Africa: An Analysis of the Current Situation’, *Institute of Medicine, Forum on Microbial Threats*, Washington DC, 24 March 2015.

75.
‘Travel Summary Report’, 8 July 2014, Associated Press *op. cit.* (note 1).

76.
Médecins Sans Frontières, *op. cit.* (note 7).

77.

http://www.who.int/csr/disease/ebola/ebola-6-months/sierra-leone/en/(accessed 17 June 2015).

78.
Wauquier *et al*, *op. cit.* (note 56).

79.

Stephen K. Gire , ‘Genomic Surveillance Elucidates Ebola Virus Origin and Transmission during the 2014 Outbreak’, Science, 345, 6202 (2014), 1369–1372.2521463210.1126/science.1259657PMC4431643

80.
Samuel A. Sheku Kargbo, interview with author, Freetown, 3 March 2015.

81.

Etienne Simon-Lorière , ‘Distinct Lineages of Ebola Virus in Guinea during the 2014 West African Epidemic’, Nature, 524, 7563 (2015), 102–104.2610686310.1038/nature14612PMC10601606

82.
Wauquier *et al*, *op. cit.* (note 56).

83.
Nadia Wauquier, interview with author, Paris, 28 May 2015.

84.
Joseph Fair, interview with author, Washington DC, 24 March 2015; Will Pooley, interview with author, Eycke, Suffolk, 24 May 2015.

85.
Joshua Hammer, ‘My Nurses are Dead and I Don’t Know if I’m Already Infected’, *Medium*, 2015, https://medium.com/matter/did-sierra-leones-hero-doctor-have-to-die-1c1de004941e#.pslijyrgh(accessed 4 February 2015).

86.

Daniel Bausch , ‘A Tribute to Sheik Humarr Khan and All the Healthcare Workers in West Africa who Have Sacrificed in the Fight against Ebola Virus Disease: Mae We Hush’, Antiviral Research, 111 (2014), 33–35.2519653310.1016/j.antiviral.2014.09.001

87.
Joel Montgomery, interview with author, CDC, Atlanta, 27 October 2015.

88.
Baize, *op. cit.* (note 1).

89.
Simon-Lorière, *op. cit.* (note 81).

90.

M. Allison Arwady , ‘Evolution of Ebola Virus Disease from Exotic Infection to Global Health Priority, Liberia, Mid-2014’, Emerging Infectious Diseases, 21, 4 (2015), 578–584: 173.2581117610.3201/eid2104.141940PMC4378496

91.
Kevin De Cock, interview with author, London, 26 June 2015.

92.
This view was cogently expressed by Oliver Morgan, who served as the CDC’s country head in Sierra Leone from November 2014 to August 2015 and was a witness to high-level discussions at the National Ebola Response Control Centre. Interview with author, CDC, Atlanta, 27 October 2015.

93.

Kevin M. De Cock and Wafaa M. El-Sadr , ‘A Tale of Two Viruses: HIV, Ebola and Health Systems’, AIDS, 29, 9 (2015), 989–991.2612513510.1097/QAD.0000000000000726

94.
James Nye and Associated Press, ‘Ebola Victim who Sparked Fears of a Global Outbreak was American’, *Mail Online*, 29 July (2014), http://www.dailymail.co.uk/news/article-2709180/Ebola-victim-sparked-fears-global-outbreak-American-Father-died-incurable-virus-Nigeria-taking-international-flight-going-visit-children-Minnesota.html(accessed 17 November 2015).

95.
Nigeria’s successful containment of secondary spread of Ebola was also heavily reliant on infrastructure and human capital investments it had received for polio eradication. De Cock and El-Sadr, *op. cit.* (note 93).

96.
Apparently, Sawyer, whose wife and children lived in Minnesota, had been intending to travel to the United States in August. Nye and Associated Press, *op. cit.* (note 94).

97.
De Cock, *op. cit.* (note 91).

98.

http://www.huffingtonpost.com/2014/08/03/donald-trump-ebola-doctors_n_5646424.html(accessed 10 November 2015).

99.
Monica Mark, ‘Ebola Outbreak “Moving Faster than Efforts to Control it”, says WHO Chief’, *The Guardian*, 1 August (2014).

100.
De Cock, *op. cit.* (note 91).

101.

http://www.un.org/ebolaresponse/response.shtml(accessed 29 November 2016).

102.
‘Report of WHO Interim Assessment Panel’, *op. cit*. (note 7).

103.
Suerie Moon *et al*, ‘Will Ebola Change the Game? Ten Essential Reforms before the next Pandemic: The Report of the Harvard-LSHTM Independent Panel on the Global Response to Ebola’, *The Lancet*, 386 (2015), 2204–21: 1009.10.1016/S0140-6736(15)00946-0PMC713717426615326

104.
‘Global Health Risk Framework, National Academy of Medicine’, 2016, https://nam.edu/initiatives/global-health-risk-framework/(accessed 5 August 2016).

105.
‘Report of WHO Interim Assessment Panel’, *op. cit*. (note 7).

